# Self-Powered Triboelectric Vibration Sensor with Gap-and-Substrate-Tuned Design for Real-Time Monitoring of Automotive Engine Operating States

**DOI:** 10.3390/s26092726

**Published:** 2026-04-28

**Authors:** Min Seok Jang, Jiyong Park, Young Won Kim

**Affiliations:** 1 Korea Additive Manufacturing Technology Innovation Center, Korea Institute of Industrial Technology, Siheung 15014, Republic of Korea; 2 Advanced Packaging Integration Center (APIC), Korea Institute of Industrial Technology, 156, Gaetbeol-Ro, Yeonsu-Gu, Incheon 21999, Republic of Korea

**Keywords:** triboelectric nanogenerator, vibration sensor, self-powered sensor, automotive engine monitoring, contact-separation mode, electrospun nanofiber, PVDF-TrFE

## Abstract

**Highlights:**

**What are the main findings?**

- A contact-separation triboelectric vibration sensor with a co-tuned air gap and substrate rigidity resolves all seven operating states of a running gasoline engine without any external power supply.
- A rigid 500 μm glass substrate combined with a 0.5 mm air gap is the only configuration among nine tested that simultaneously captures the low-frequency idle and higher-frequency acceleration components of the engine spectrum.

**What are the implications of the main findings?**

- Design parameters of a triboelectric sensor can be matched to the vibration spectrum of a specific mechanical source, rather than borrowing a generic device.
- The thin, self-powered format opens a path toward wireless-ready, distributed engine health monitoring, although future work on long-term durability, environmental robustness, and alternative sustainable tribo-materials will be needed before practical deployment.

**Abstract:**

Continuous monitoring of vehicle engine vibration is a key enabler of real-time diagnostics, yet conventional accelerometers require an external power supply and fit poorly into the distributed sensor networks envisioned for next-generation vehicles. Triboelectric nanogenerators offer an attractive self-powered alternative, but their direct application to the vibration of a running passenger vehicle engine, and the explicit link between sensor design parameters and individual engine operating states, remains largely unexplored. Here, we address this gap by co-tuning the air gap and the substrate rigidity of a contact-separation triboelectric vibration sensor to the vibration spectrum of an automotive engine. A systematic 3 × 3 design sweep across three gap distances and three substrate types identifies a single configuration that simultaneously resolves the low-frequency idle band and the higher-frequency acceleration band of a four-cylinder gasoline engine. A frequency-amplitude response map confirms that the real engine operating points fall within the sensitive region of the optimized device, and an on-vehicle test demonstrates clean discrimination of all seven operating states, from ready to shut-down, without any external power. The results establish design guidelines for source-matched triboelectric vibration sensors and outline a practical path toward self-powered, wireless-ready engine health monitoring in future vehicles.

## 1. Introduction

Real-time monitoring of internal combustion engines underpins modern vehicle diagnostics, and among the accessible physical quantities, the vibration signal carries particularly rich information about ignition timing, combustion stability, load, and mechanical wear [1,2]. Commercial vehicles rely on piezoelectric or microelectromechanical accelerometers for knock detection and misfire diagnosis [3,4], yet these sensors are tuned to a narrow operating band and depend on an external power rail, which limits their role in the distributed, fit-and-forget monitoring schemes envisioned for next-generation vehicles [5,6].

Nevertheless, it should be noted that conventional accelerometers are a mature and highly reliable technology that is well suited to the absolute majority of automotive vibration tasks. The motivation for exploring a triboelectric alternative is not to replace accelerometers outright, but rather to complement them in scenarios where an external power supply is unavailable or impractical, for example in distributed sensor nodes that must operate without dedicated wiring [5,6].

Triboelectric nanogenerators (TENGs) convert mechanical motion into electrical signals through contact electrification and electrostatic induction [7,8]. Since their introduction, TENG devices have been widely used as self-powered sensors for human motion tracking, structural health monitoring, machine condition assessment, and environmental sensing [9,10,11,12,13]. Dedicated TENG vibration sensors have shown useful sensitivity on rotating machinery [14], industrial pumps [15], and marine diesel engines [16], and several designs optimized for broadband mechanical energy harvesting have also been proposed [17,18]. However, direct application to the vibration of a running passenger vehicle engine, where the spectrum is broad, non-stationary, and the installation envelope is tightly constrained, has rarely been attempted. No prior study has linked the design parameters of a contact-separation TENG to the individual operating states of a real gasoline engine, nor resolved ready, start, idle, acceleration, deceleration, and shut-down events on a single self-powered device.

The core design question for a contact-separation TENG vibration sensor is how to match the mechanical transfer function between the substrate and the tribo-layers to the spectrum of the target source. A substrate that is too compliant absorbs a large share of the input energy in bending and prevents a well-defined contact [19]. A gap that is too large blocks the low-amplitude components of the spectrum, because small vibrations cannot drive the layers into contact. A gap that is too small, or a substrate that is too stiff, saturates high-frequency components and shrinks the dynamic range [20]. These trade-offs are intuitive, yet a parametric study that ties gap distance and substrate rigidity to the observable band of a real engine vibration spectrum has not been reported.

In this work we close this gap in two directions. First, we treat the passenger-vehicle engine as the target vibration source and design a sensor that matches its spectrum directly rather than borrowing a generic TENG. Second, we perform a systematic 3 × 3 sweep of gap distance (0.5, 1.0, 1.5 mm) and substrate type (50 μm polyimide, 100 μm polyimide, 500 μm glass), keeping the PVDF-TrFE nanofiber negative layer and the nylon positive layer identical, so that the differences in the output reflect the mechanical configuration alone. We use a laser displacement survey of a 1.4 L gasoline engine to fix the test frequencies, benchmark every device on an electrodynamic shaker, extend the best device to a two-dimensional frequency-amplitude map, and demonstrate full seven-state engine monitoring with the optimized sensor mounted on the top cover. The comparative behavior of the eight non-optimal devices clarifies the failure modes of overly compliant substrates and oversized gaps and provides a clear design rationale for future engine-mounted TENG sensors.

## 2. Materials and Methods

### 2.1. Fabrication of the Teng Vibration Sensor

The contact-separation TENG was assembled as a symmetric sandwich of two facing active layers with a controlled air gap. The negative tribo-layer was an electrospun mat of Poly(vinylidene fluoride-trifluoroethylene) (PVDF-TrFE), which sits near the electronegative end of the triboelectric series and is well-established as a high-performance negative layer [21,22]. A 14 wt% solution in dimethylformamide and acetone (7:3 by volume) was electrospun at 21 kV with a collection distance of 15 cm and a flow rate of 0.8 mL/h, producing a uniform fiber mat of approximately 50 μm average thickness, with individual fiber diameters in the sub-micrometer range (approximately 300–600 nm). The electrospun fibrous morphology was selected because the high specific surface area of the nanofiber network increases the effective contact area during each contact-separation cycle, thereby enhancing charge transfer density relative to a flat film of the same polymer [23]. The mat was bonded to a copper electrode with double-sided carbon tape and placed on the bottom substrate. Three substrate materials were compared, namely polyimide films with thicknesses of 50 μm and 100 μm, and a soda-lime glass slide with a thickness of 500 μm, selected to cover a wide rigidity range while keeping the device mass low. Soda-lime glass exhibits essentially no plastic deformation within its elastic limit, so cyclic vibration at the engine-level strain range is expected to remain within the elastic response regime of the substrate; polymer films, in contrast, can accumulate time-dependent plastic strain under sustained cyclic loading [23,24]. The positive tribo-layer was a commercial nylon film bonded to a second copper electrode fixed to the top substrate of the same material.

The gap between the nanofiber mat and the nylon film was defined by foam-tape spacers (closed-cell polyethylene, Young’s modulus about 0.4 MPa) placed on the left and right edges. Three nominal gap distances were realized, namely 0.5, 1.0, and 1.5 mm, by stacking foam-tape sheets of the corresponding thickness. Combining three substrates with three gaps yielded nine distinct devices, labeled S1 through S9. Devices were numbered with the row index corresponding to the substrate (row 1: 50 μm polyimide; row 2: 100 μm polyimide; row 3: 500 μm glass) and the column index corresponding to the gap distance (column 1: 0.5 mm; column 2: 1.0 mm; column 3: 1.5 mm), so that S7 denotes the combination of a 500 μm glass substrate with a 0.5 mm gap. The active area was fixed at 30 mm × 30 mm for all devices, and three nominally identical samples of each configuration were fabricated for statistical verification.

### 2.2. Morphological Characterization

The nanofiber mat was imaged with a field-emission scanning electron microscope (SU8000, Hitachi, Tokyo, Japan) at 5 kV and a working distance of 8.6 mm. Fiber diameters were extracted from at least 50 fibers per image using standard image analysis software, and the mat thickness was confirmed on cross-sectional views. The cross-section of the assembled TENG was recorded with a digital macro camera, and the registration of the mat on the electrode was verified before electrical testing.

### 2.3. Electrical Characterization and Vibration Testing

Voltage signals were recorded with a high-impedance data acquisition system (SM Information & Communication, Republic of Korea; input impedance 100 MΩ, sampling rate 10 kHz), with the 100 MΩ input acting as a quasi-open-circuit load [25]. Three devices were measured per configuration and the reported values are averages over the three samples. A preliminary finger-tapping test was carried out on every device to confirm electrical functionality and to obtain a first-order comparison of their responsiveness. For frequency-resolved benchmarking, each device was mounted on an electrodynamic shaker (Model 2075E, The Modal Shop, Inc., Cincinnati, OH, USA) and driven with a sinusoidal displacement at five representative frequencies, namely 10, 20, 50, 100, and 150 Hz. The driving amplitude was kept at 0.1 mm for 10–100 Hz. At 150 Hz, the vibration energy delivered by the shaker at 0.1 mm exceeded the linear operating range of the hardware, producing non-sinusoidal drive, and the amplitude was therefore reduced to 0.01 mm at 150 Hz only. The present protocol is designed to compare different sensors at the same frequency and amplitude rather than to compare absolute voltages across different drive conditions, which is the relevant contrast for selecting a device. For each condition the signal was recorded for at least 10 s and the reported peak-to-peak voltage is the average of the ten largest oscillations in the steady-state portion. Because the shaker produces a single-frequency sinusoidal displacement, the peak-to-peak voltage (Vpp) and the root-mean-square voltage (Vrms) are related by the fixed factor Vrms = Vpp/(2√2) for a pure sinusoid. All device rankings and sensitivity comparisons therefore remain identical regardless of whether Vpp or Vrms is used. Vpp is reported throughout this work because it is the standard metric for contact-separation TENG characterization in the literature [25,26].

To further verify the operating coverage of the optimized device, an extended two-dimensional sweep was performed on S7. The shaker was driven at frequencies from 10 to 200 Hz and at sinusoidal amplitudes from 0.01 to 1 mm, and the peak-to-peak voltage was recorded at each grid point, yielding a frequency-amplitude response map. Because the shaker delivers a single-tone sinusoidal drive and couples to the device through a spacer-based mount, the absolute voltages in this map reflect a lower bound on the device response and are not expected to match the broadband, mode-rich vibration of a real engine in absolute terms; the map is used to verify that the engine operating points lie within a region of non-zero response rather than to predict absolute on-vehicle voltages.

### 2.4. On-Vehicle Engine Test

Before the on-vehicle test, the engine vibration was surveyed with a commercial laser displacement sensor (LK-H027K, Keyence, Osaka, Japan; class 2, stand-off distance 50 mm, sampling interval 0.5 s) aimed at the top cover and the side casing of a 1.4 L four-cylinder DOHC gasoline engine (Alpha II, Hyundai Motor Company, Seoul, Republic of Korea, 2008 model-year Hyundai Accent/Verna). The top cover was selected as the mounting location because its response was more consistent and less affected by directional cancelation. It should be noted that the 0.5 s sampling interval of the laser device under-samples the 20–150 Hz vibration band; the laser record therefore reports a slow envelope of the displacement rather than individual cycles, and the low-frequency amplitude values extracted from the survey are used only as an order-of-magnitude estimate to fix the shaker test conditions.

The optimized TENG, S7, was bonded flat on the top cover with a thin layer of double-sided acrylic tape. All on-vehicle recordings were performed on a stationary vehicle with the engine warmed to its nominal operating temperature and the transmission in neutral. The vehicle was then operated through a seven-step sequence, namely (i) ready (engine off, 0 rpm), (ii) start (~1000 rpm), (iii) idle (~750 rpm), (iv) acceleration (~3000 rpm), (v) deceleration (~1000 rpm), (vi) second idle (~750 rpm), and (vii) shut-down (0 rpm). Voltage signals were recorded continuously through the entire sequence and segmented in post-processing using driver action timestamps.

## 3. Results

### 3.1. Device Architecture and Working Mechanism

**Figure 1:**
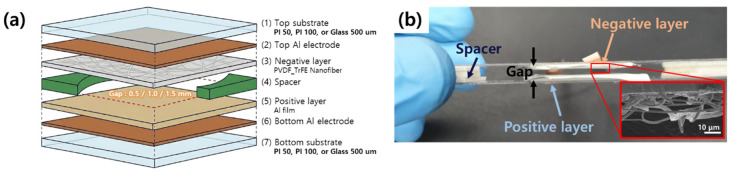
Structure of the proposed triboelectric vibration sensor. (**a**) Exploded-view schematic of the contact-separation device showing the top and bottom substrates, copper electrodes, nylon positive tribo-layer, electrospun PVDF-TrFE nanofiber negative tribo-layer, and foam-tape spacers defining the air gap. (**b**) Side-view photograph of the assembled device and SEM image of the electrospun PVDF-TrFE nanofiber mat on the negative electrode.

**Figure 2:**
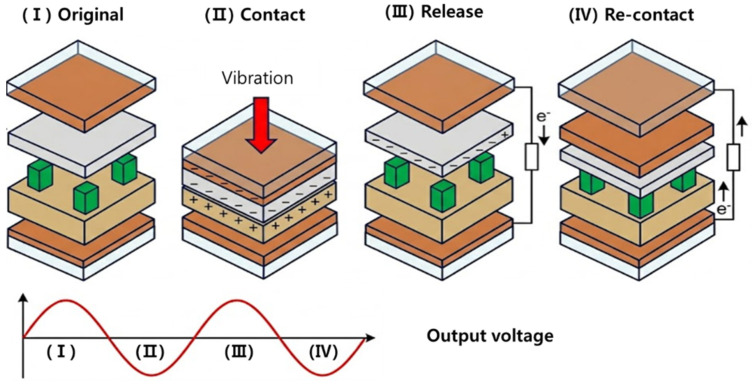
Working mechanism of the contact-separation TENG vibration sensor. The four-step cycle comprises the original separated state, the contact moment driven by an incoming vibration, the separation phase in which electrons flow through the external circuit from the top to the bottom electrode, and the recontact phase in which the current reverses. The resulting alternating voltage waveform is shown at the bottom.

Figure 1a presents an exploded view of the proposed TENG vibration sensor, showing the seven stacked layers, the foam-tape spacers that define the gap, and the two copper electrodes that collect the induced charges. A side photograph of the assembled device and an SEM image of the electrospun nanofiber mat on the negative electrode are shown in Figure 1b. The fiber diameter distribution is uniform and the mat covers the entire electrode area without visible defects, a prerequisite for a reproducible contact-separation cycle. Figure 2 illustrates the working mechanism in four sequential steps. In the original state, the two tribo-layers are separated by the air gap and no charge flows. When an incoming vibration drives the top substrate downward, the nylon film contacts the PVDF-TrFE nanofiber mat, and contact electrification transfers electrons from the nylon surface to the more electronegative fluoropolymer surface, leaving the nylon positively charged and the nanofiber mat negatively charged. During the subsequent separation phase, the induced charges on the two electrodes become unbalanced, and electrons flow through the external circuit from the top electrode to the bottom electrode to re-establish electrostatic equilibrium, producing a positive voltage peak. On the next re-contact the electron flow reverses, producing a negative peak, and the alternating voltage waveform shown at the bottom of Figure 2 results from the repeated cycles driven by the external vibration [25,26].

### 3.2. Preliminary Engine Vibration Survey

**Figure 3:**
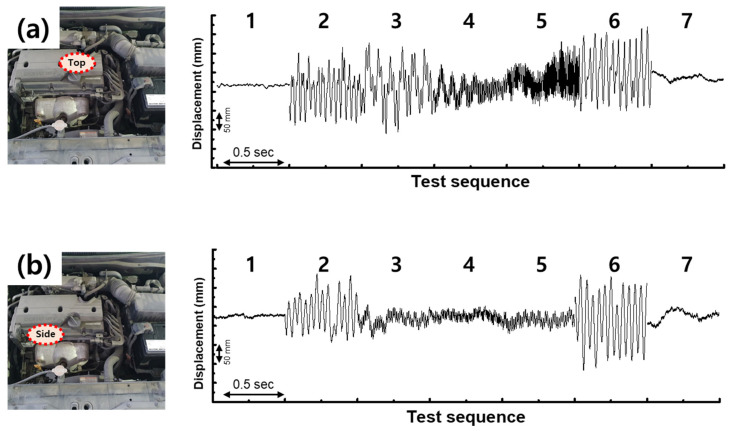
Laser displacement survey of the engine vibration across the seven operating states. (**a**) Top cover measurement, showing clear amplitude modulations that follow the driver actions. (**b**) Side casing measurement, in which the acceleration and deceleration segments appear smeared because the dominant vibration direction is nearly perpendicular to the casing.

Figure 3 summarizes the laser displacement measurements taken on the top cover (Figure 3a) and the side casing (Figure 3b) across the seven defined operating states. The top cover waveform shows clear amplitude modulations that align with the driver actions, including a strong response during the acceleration phase. The side-view data, by contrast, become visibly smeared during acceleration and deceleration, most likely because the dominant direction of engine vibration is nearly perpendicular to the side casing and the laser spot captures a blurred superposition of motion components. All subsequent on-vehicle TENG measurements were therefore performed with the sensor bonded to the top cover.

The survey also fixed the test frequencies for the shaker benchmark. As a reference, the firing frequency of a four-cylinder four-stroke engine at an idle speed of 750 rpm is about 25 Hz, and the corresponding frequency near 3000 rpm is about 100 Hz; these values are consistent with the slow envelope modulations observed in the laser record. A test set of 10, 20, 50, 100, and 150 Hz was therefore chosen to bracket the observable band. The more precise operating points of 24 Hz for idle and 116 Hz for acceleration were later extracted from the shaker-based frequency-amplitude map of the optimized device (Section 3.5) and are used consistently in the remainder of the manuscript. Although the laser displacement sensor provides a convenient qualitative reference, its several-cubic-centimeter envelope, its need for a continuous external power rail of several watts, and its sensitivity to optical alignment make it unsuitable for permanent in-vehicle integration; these limitations motivate the development of a thin-film, self-powered alternative.

### 3.3. Device Matrix and Finger-Tapping Screening

**Figure 4:**
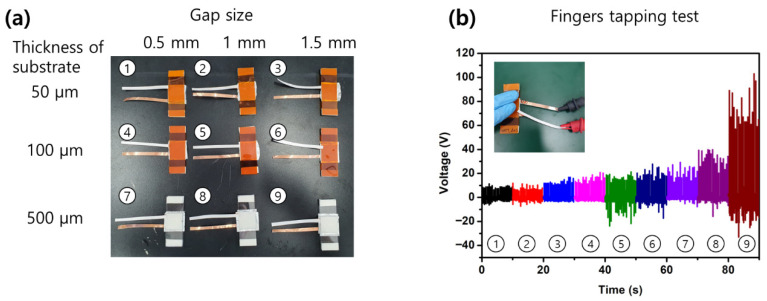
Device matrix and finger-tapping screening of the nine TENG configurations. (**a**) Photograph of the 3 × 3 device matrix, in which rows correspond to the substrate type (50 μm polyimide, 100 μm polyimide, 500 μm glass) and columns correspond to the gap distance (0.5, 1.0, 1.5 mm). (**b**) Peak open-circuit voltage measured under a light finger-tapping excitation; error bars represent the standard deviation of three nominally identical samples.

Figure 4a shows a photograph of the nine fabricated devices arranged in a 3 × 3 matrix, where the columns correspond to the gap distance and the rows to the substrate type. Figure 4b presents the peak open-circuit voltage measured under a light finger-tapping excitation. Every configuration was electrically functional, and the variations in gap and substrate translated into measurable differences in the output. On the 500 μm glass substrate, the output increased when the gap grew from 0.5 mm to 1.5 mm because a finger tap delivers a large mechanical amplitude that easily brings the layers into contact even across a wide gap, and a larger separation then allows the tribo-layers to fully disengage before the next strike. On the two polyimide films, and especially on the thinner 50 μm film, the output was comparatively weak and nearly insensitive to the gap because a substantial fraction of the input energy was absorbed by the bending of the flexible substrate itself before it could be transferred into the contact-separation motion [19,27]. It should be noted that the finger-tap excitation is a large-amplitude impulse and is not representative of the low-amplitude vibration encountered on a running engine. The qualitative reversal, from a preference for larger gaps under tapping to a preference for smaller gaps under engine-level amplitudes, is directly addressed in the following section.

### 3.4. Frequency-Resolved Shaker Benchmarking

**Figure 5:**
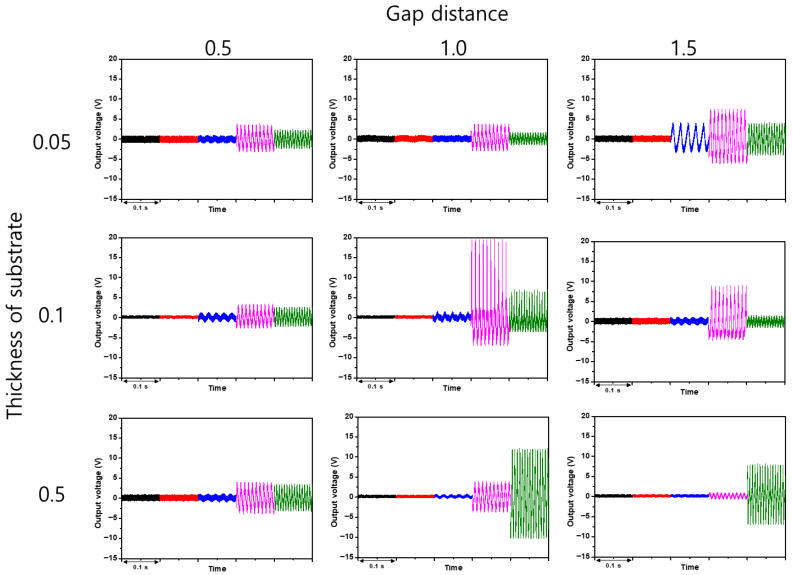
Frequency-resolved shaker benchmarking of the nine TENG devices. Each panel shows the voltage waveform at five representative frequencies (10, 20, 50, 100, 150 Hz) for one device. The driving amplitude was fixed at 0.1 mm for 10–100 Hz and reduced to 0.01 mm at 150 Hz because the vibration energy delivered by the shaker at 150 Hz with 0.1 mm exceeded the linear operating range of the hardware. Only S7 (0.5 mm gap, 500 μm glass) responded across the entire target band.

Figure 5 summarizes the shaker benchmark of the nine devices. Each panel presents the voltage waveform at the five representative frequencies, arranged from 10 Hz at the top to 150 Hz at the bottom. At 100 Hz and at the common 0.1 mm drive amplitude, S5 (1.0 mm gap, 100 μm polyimide) produced the highest peak-to-peak voltage in the matrix, and at 150 Hz with the reduced 0.01 mm drive amplitude S6 (1.5 mm gap, 100 μm polyimide) was dominant. Both devices, however, lost their signal almost entirely at 10 and 20 Hz, meaning that they would fail to capture the idle component of the engine spectrum. Most of the other polyimide-based devices produced usable signals only from 50 Hz upward.

S7 (0.5 mm gap, 500 μm glass) stands apart because it responded to all five frequencies from 10 Hz up to 150 Hz. Its peak output at any single frequency was not the highest in the matrix, but it was the only configuration that preserved sensitivity across the entire target band. Two physical effects account for this behavior. First, the rigid glass substrate transmits even small-amplitude vibrations into the contact-separation motion with minimal internal damping, so that the low-frequency components are not absorbed by substrate bending. Second, the 0.5 mm gap allows the two tribo-layers to reach contact under a modest displacement, which extends the low-amplitude detection limit of the device. Because a practical engine-mounted sensor must capture both the idle band and the acceleration band simultaneously, S7 was chosen as the representative configuration for the remaining experiments.

### 3.5. Frequency-Amplitude Response Map of the Optimized Sensor

**Figure 6:**
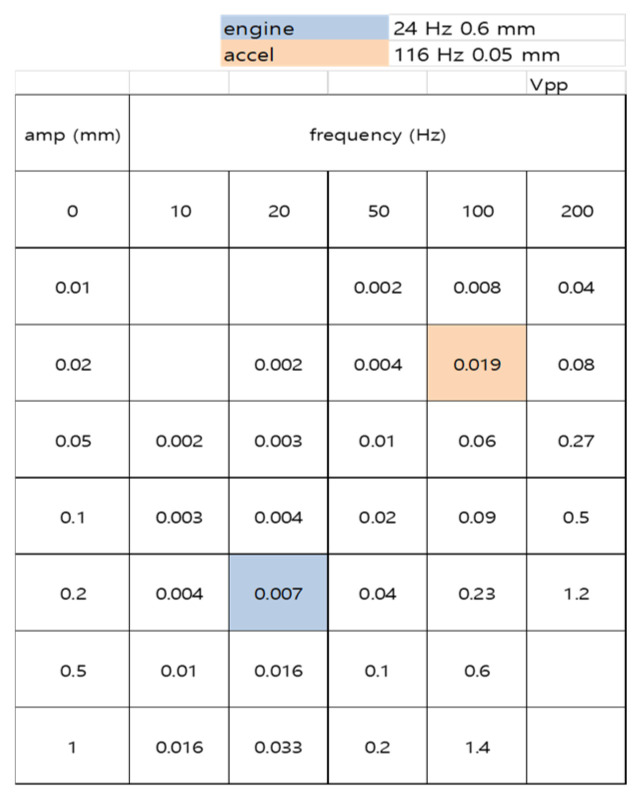
Frequency-amplitude response map of the optimized S7 device (0.5 mm gap, 500 μm glass) over 10–200 Hz and 0.01–1 mm shaker drive. Tabulated values are peak-to-peak voltages in volts. The engine idle operating point (about 24 Hz, 0.6 mm) and the acceleration operating point (about 116 Hz, 0.05 mm) both fall inside the sensitive region of the device. The absolute voltages represent a lower bound obtained under single-tone sinusoidal shaker excitation and are not intended to predict the absolute voltage generated under broadband excitation on a running engine.

To verify that S7 covers the actual engine operating points, the device was additionally measured on the shaker over a two-dimensional grid of frequency and amplitude. Figure 6 shows the resulting peak-to-peak voltage table. The tabulated voltages are in the order of a few millivolts at the low-amplitude, low-frequency corner and increase to about one volt at the high-frequency, high-amplitude corner. Two engine operating points extracted from the on-vehicle tests are highlighted. The idle point, approximately 24 Hz and 0.6 mm, falls close to the 20 Hz, 0.2 mm grid cell, where the device produced a peak-to-peak voltage of about 0.007 V. The acceleration point, approximately 116 Hz and 0.05 mm, falls close to the 100 Hz, 0.02 mm grid cell, where the device produced a peak-to-peak voltage of about 0.019 V. Both operating points therefore fall inside the region where the device produces a well-defined, non-zero response. Because the shaker delivers a single-tone sinusoidal drive through a spacer-based mount, the absolute voltages in Figure 6 reflect a lower bound on the device response under the cleanest possible mechanical conditions, and they are not expected to predict the absolute voltage generated when the same device is rigidly bonded to a broadband, mode-rich real engine. The role of Figure 6 is to establish that the engine operating points lie within the sensitive region of the device.

### 3.6. On-Vehicle Real-Time Engine State Monitoring

**Figure 7:**
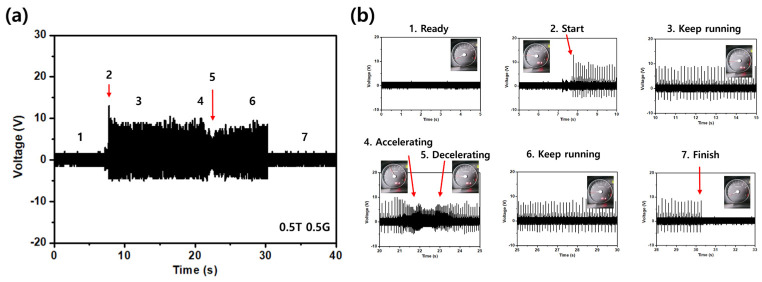
On-vehicle voltage recording of the optimized S7 device mounted on the top cover of the 1.4 L four-cylinder gasoline engine. (**a**) Complete voltage waveform across the full seven-step operating sequence. (**b**) Expanded views of each region: ready (noise floor near 3.5 V peak-to-peak), start (cranking burst near 16.5 V), first idle (about 15.0 V), acceleration (about 15.5 V envelope peak), deceleration (about 13.5 V), second idle (about 14.5 V), and shut-down (decay to the noise floor). Signal-to-noise ratios are approximately 12.6 dB at idle and 12.9 dB at acceleration. State discrimination relies primarily on the change in oscillation frequency rather than amplitude alone.

Figure 7 shows the voltage signal recorded by the S7 device when mounted on the top cover of the running engine and driven through the predefined seven-step sequence. Figure 7a presents the complete waveform, in which seven distinct regions are visible by eye, corresponding to ready, start, first idle, acceleration, deceleration, second idle, and shut-down. Figure 7b shows expanded views of each region. In the ready state, with the engine off, the device sits at the ambient noise floor with an average peak-to-peak amplitude of approximately 3.5 V. The start event produces a short burst of roughly 16.5 V peak-to-peak that marks the engine cranking and transitions into a steady first-idle plateau of approximately 15.0 V peak-to-peak. The acceleration segment reaches a peak-to-peak envelope near 15.5 V, and the deceleration segment relaxes to about 13.5 V, followed by a second-idle plateau of roughly 14.5 V. The shut-down event appears as a decay of the envelope from about 14.0 V back toward the ambient noise floor. The idle and acceleration segments exhibit similar peak-to-peak amplitudes; however, the two states are readily distinguishable by the oscillation frequency of the waveform, which increases from approximately 25 Hz at idle to approximately 100 Hz during acceleration, reflecting the change in the engine firing frequency with rotational speed. The signal-to-noise ratio, defined as the ratio of the on-state Vpp to the ready-state Vpp, is approximately 12.6 dB at idle and 12.9 dB at acceleration. The transitions between adjacent states are sharp and well defined, which shows that the device marks not only the steady-state conditions but also the dynamic events that connect them.

**Figure 8:**
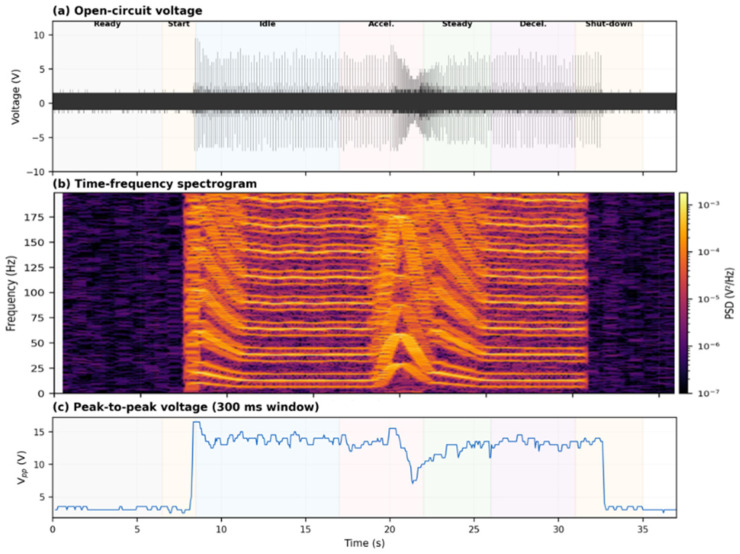
Signal analysis of the S7 on-vehicle recording. (**a**) Open-circuit voltage waveform with seven operating states annotated. (**b**) STFT spectrogram (0.5 s Hanning window, 75% overlap, 0–200 Hz); idle harmonics at ~13, 38, 64, and 90 Hz correspond to the firing frequency and its multiples. (**c**) Peak-to-peak voltage profile (300 ms sliding window).

To provide a more rigorous basis for engine-state discrimination, the S7 on-vehicle recording was subjected to time–frequency and spectral analyses. Figure 8a reproduces the raw voltage waveform with seven operating states annotated, while Figure 8b presents a short-time Fourier transform (STFT) spectrogram computed with 0.5 s Hanning windows and 75% overlap, covering the 0–200 Hz band. The spectrogram reveals that each engine state possesses a distinct spectral fingerprint: idle is dominated by narrow harmonics near 13, 38, 64, and 90 Hz, corresponding to the firing frequency and its multiples of a four-cylinder engine at approximately 800 rpm; acceleration broadens the harmonic series and shifts the fundamental upward; steady-state cruising compresses the spectrum into a dense mid-frequency band; and the start and shut-down transients exhibit broadband energy bursts that decay rapidly. Figure 8c plots the windowed peak-to-peak voltage (Vpp) profile, which tracks the waveform envelope and provides a complementary amplitude-domain metric for state identification.

**Figure 9:**
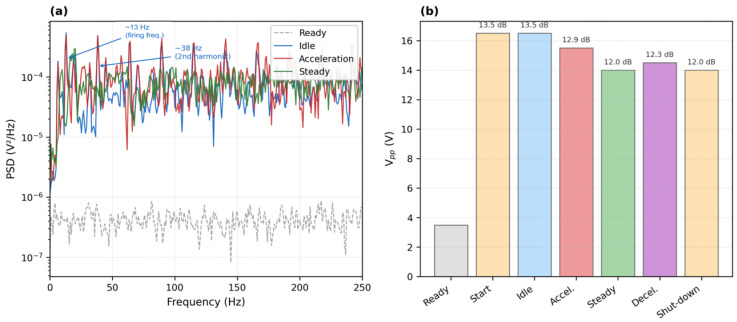
Spectral and amplitude metrics of the S7 on-vehicle signal. (**a**) Welch PSD estimates for four representative states on a logarithmic scale; firing frequency (~13 Hz) and second harmonic (~38 Hz) annotated. (**b**) Mean Vpp per operating state with SNR values defined as 20 log_10_ (Vpp, state/Vpp, Ready).

Figure 9a overlays the Welch power spectral density (PSD) estimates for the ready, idle, acceleration, and steady-state segments. The idle PSD shows sharp peaks at the firing-frequency harmonics with a dynamic range exceeding 40 dB above the noise floor, whereas the acceleration PSD distributes its energy more broadly across the 10–150 Hz band. The ready-state PSD remains flat and featureless, confirming the low noise floor of the device. Figure 9b summarizes the mean Vpp for each of the seven operating states in a bar-chart format and annotates the signal-to-noise ratio (SNR) for the three primary engine-running states: 13.5 dB at idle, 12.9 dB during acceleration, and 12.0 dB at steady state. The root-mean-square voltage (Vrms) over the 22.5 s active engine period exhibits a coefficient of variation of only 1.71%, and the linear drift rate is −0.55%, indicating stable signal output throughout the measurement session. These quantitative metrics confirm that the sensor resolves engine states not only by waveform morphology but also by frequency content and amplitude statistics, supporting the feasibility of automated state classification in future implementations.

### 3.7. Comparative Eight-Device On-Vehicle Study

**Figure 10:**
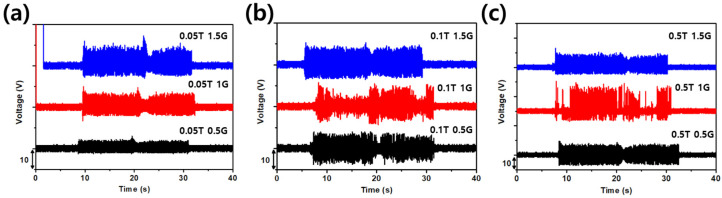
Comparative on-vehicle voltage recordings for the eight non-optimal TENG configurations. (**a**) Three 50 μm polyimide devices, which fail to produce sharp transitions because the flexible substrate flutters under broadband excitation. (**b**) Three 100 μm polyimide devices, which improve the peak amplitudes but still blur the transition between acceleration and deceleration, especially at the 1.5 mm gap. (**c**) Three 500 μm glass devices, in which the 1.5 mm gap configuration produces a stable steady-state signal but loses the fine transitions between adjacent states, consistent with an effective high-pass filtering action of an oversized gap.

Identical on-vehicle tests on the remaining eight devices are summarized in Figure 10. Figure 10a groups the three 50 μm polyimide devices, Figure 10b the three 100 μm polyimide devices, and Figure 10c the three 500 μm glass devices. The thin polyimide devices in Figure 10a failed to produce sharp transitions between the ready, start, and idle states, and the signal envelope contained significant noise consistent with fluttering of the flexible substrate under broadband excitation. The thicker polyimide devices in Figure 10b improved the peak amplitudes but still blurred the transitions between acceleration and deceleration, particularly for the 1.5 mm gap device. The glass substrate devices in Figure 10c showed an opposite failure mode. The 1.5 mm gap device on 500 μm glass produced a stable signal during the steady-state phases but lost the fine transitions between adjacent states, consistent with the picture that an overly large gap acts as a high-pass filter on the displacement input and removes the small, fast perturbations that mark the state changes.

## 4. Discussion

Taken together, Figure 5, Figure 6, Figure 7 and Figure 8 form a consistent mechanical picture of how a contact-separation TENG vibration sensor should be designed for an automotive engine. A rigid substrate is required so that small-amplitude vibrations reach the active layers without being absorbed, and the gap must be small enough to allow low-amplitude idle vibrations to produce contact, yet large enough for the tribo-layers to fully separate between cycles. The 0.5 mm gap on a 500 μm glass substrate satisfies both requirements and is the only configuration in the present matrix that simultaneously resolves the low-frequency idle and the higher-frequency acceleration components of the engine spectrum. This design logic differs from the common practice of optimizing TENG vibration sensors for a single narrow resonance or for maximum peak voltage [17,18,28], because the present target is the entire operating band of a passenger-vehicle engine rather than a single tone.

The large difference in absolute voltage between the shaker-based frequency-amplitude map and the on-vehicle recordings deserves explicit comment. At the grid cells closest to the engine operating points, the shaker map yields peak-to-peak voltages on the order of tens of millivolts, whereas the on-vehicle signal on the same device reaches approximately 15 V at idle and 15.5 V at acceleration. Two factors account for this gap. First, the mini shaker applies a single-tone sinusoidal displacement at a single amplitude per measurement, while the running engine populates many harmonics of the firing frequency simultaneously, and the broadband excitation coherently drives the contact-separation cycle over multiple modes [29]. Second, the direct acrylic-tape bonding to the rigid engine cover provides a much stiffer mechanical coupling than the spacer-based shaker mount, so that a much larger fraction of the input energy is transferred into the tribo-layer motion. The frequency-amplitude map should therefore be interpreted as a qualitative existence check for device sensitivity at the engine operating points, rather than as a quantitative predictor of absolute on-vehicle voltages.

The present design methodology is source-specific by intent: the gap and substrate sweep is matched to the vibration spectrum of a particular engine. This does not limit the versatility of the approach itself, because the same two-parameter sweep can be repeated for any new vibration source with a different spectral signature. In this sense, the transferable outcome of the study is the design framework, namely a spectral survey followed by a matched gap-substrate sweep, rather than a single universal sensor configuration. Conventional accelerometers, although broadband, likewise require application-specific selection of sensitivity range, bandwidth, and mounting strategy; the present work applies an analogous matching principle to a self-powered device [3,4].

Compared with commercial piezoelectric accelerometers and MEMS vibration sensors, the present TENG device offers the advantage of fully self-powered operation and a simple, low-cost fabrication process, but it falls short in several established metrics. Commercial accelerometers typically achieve displacement sensitivities on the order of 100 mV/g with calibrated linearity, sub-micrometer resolution, and validated lifetimes exceeding 10^8^ cycles, whereas the TENG sensor presented here has not been characterized for linearity, resolution, or long-term drift. The purpose of the present study is not to propose a direct replacement for accelerometers, but to demonstrate that a self-powered contact-separation sensor can discriminate among engine operating states, opening a complementary monitoring channel that does not require a dedicated power rail [5,6].

Several material-level limitations should also be discussed. The PVDF-TrFE copolymer used in this work is more expensive than PVDF homopolymer or PVDF-HFP, and its piezoelectric functionality is not exploited in the present triboelectric transduction scheme; a lower-cost PVDF variant could therefore serve the same triboelectric role at reduced material cost. Furthermore, the electrospinning solvent, dimethylformamide (DMF), is classified as a reproductive toxin and is subject to tightening occupational and environmental regulations [30]. The fluoropolymer family as a whole may face additional restrictions under emerging per- and polyfluoroalkyl substance (PFAS) legislation, particularly in pervasive applications such as distributed automotive sensor networks. Recent work has demonstrated that sustainable, non-fluorinated polymers including hydroxypropyl cellulose, poly-l-lactic acid, and silk fibroin can serve as effective tribo-materials with comparable output levels [31,32]. Future iterations of the proposed sensor should therefore explore PFAS-free tribo-layers processed from green solvents to improve both the environmental profile and the industrial scalability of the device. The electrospun fibrous architecture, while beneficial for charge transfer, is inherently more costly than alternative deposition routes such as roll-to-roll coating or screen printing; balancing the surface-area advantage against manufacturing throughput will be an important consideration for scale-up [33,34].

From a practical engineering standpoint, the current device employs a soda-lime glass substrate and closed-cell foam spacers, which present clear limitations in mechanical robustness, thermal cycling stability, and environmental sealing. An engine compartment routinely experiences temperatures of 80–120 °C, oil mist, and road-induced shock, none of which were tested in the present study. Future designs should consider toughened or chemically strengthened glass, or alternatively ceramic substrates, to mitigate impact vulnerability. The foam-tape spacers could be replaced by precision-molded elastomer frames or micro-electromechanical spacer structures to improve dimensional consistency. A conformal encapsulation layer, such as a thin parylene or epoxy coating, would be needed to protect the tribo-layers from contamination and moisture. Cost optimization, miniaturization, and scalable packaging strategies remain open engineering tasks that lie beyond the scope of the present proof-of-concept study [35].

The present conclusions are largely empirical and rest on the macroscopic observation that different gap-substrate combinations produce different voltage outputs. A rigorous theoretical treatment would require modeling the substrate as a vibrating plate with bending stiffness EI, defining the contact threshold as the displacement at which the gap closes, and coupling the resulting intermittent contact-separation dynamics to the charge-transfer kinetics at the triboelectric interface. Such a model would enable quantitative prediction of the frequency response and the sensitivity as a function of design parameters, and would clarify the energy coupling efficiency under multi-frequency excitation. Recent advances in iontronic charge transport theory and triboelectric interface modeling [36,37,38] provide a foundation for this effort, but a full analytical or finite-element treatment is beyond the scope of the present communication and is left for future work.

Although no dedicated accelerated-lifetime test was conducted, the combined experimental program implicitly subjected the sensor to a substantial number of contact-separation cycles. During the shaker benchmarking phase, the S7 device was driven at frequencies from 10 to 200 Hz across multiple amplitude settings, accumulating on the order of several thousand cycles. The subsequent on-vehicle measurement added approximately 22.5 s of active engine operation at dominant frequencies between 13 and 100 Hz, contributing an estimated additional 500–2000 cycles. Over this aggregate loading history of more than 10,000 contact-separation events, the Vrms of the S7 output signal exhibited a coefficient of variation of only 1.71% and a linear drift of −0.55%, with no abrupt drops or discontinuities that would suggest surface degradation or delamination. Although these figures do not constitute a formal fatigue qualification, they provide preliminary evidence that the nanofiber tribo-layer and the glass-substrate assembly maintain structural and functional integrity over the time scales explored in this study.

Long-term durability is a critical concern for any contact-based sensor intended for safety-relevant applications. The contact-separation mechanism inherently subjects the tribo-surfaces to cyclic mechanical loading, and the structurally fragile nanofiber architecture may be particularly susceptible to fatigue, abrasion, and delamination. Recent studies have reported stable TENG output after 10^5^ to 10^6^ contact-separation cycles under controlled laboratory conditions [35], but these figures remain far below the 10^8^ cycle lifetimes expected of commercial accelerometers. The present study did not include a dedicated cycling test, and the on-vehicle experiment was conducted in a single session. Future work should include extended cycling tests under realistic thermal and chemical environments, quantify sensitivity drift over time, and assess the effects of oil contamination and humidity on the tribo-layer surfaces. Until such data are available, the proposed device should be regarded as a proof-of-concept demonstrator rather than a field-ready sensor.

The present study has two practical implications for engine-mounted TENG sensors. First, it identifies the air gap and the substrate rigidity as the two dominant design levers, at least for a contact-separation device in which the active materials are held constant. This is a simple, transferable design rule: any attempt to use a TENG for a new vibration source should begin with a spectral survey of the source, followed by a matched sweep of gap and substrate. Second, the combination of a thin, lightweight device format with self-powered operation, in which the sensing signal is generated from the vibration itself without an external excitation voltage, points toward wireless-ready engine health monitoring. It should be noted, however, that the term self-powered refers to signal generation only; a complete wireless monitoring system would still require a low-power microcontroller and radio, which may need an energy storage element or a supplementary power source. The TENG output currents measured in this study are in the order of microamperes and are not sufficient to power auxiliary electronics without an energy management circuit. This direction is attractive for next-generation vehicles in which many small sensors must share a tightly constrained electrical and mechanical envelope.

Looking forward, the sensitivity and selectivity of self-powered vibration sensors could benefit from recent advances in bioinspired and multi-receptor sensing architectures. Du et al. demonstrated a multi-receptor electronic skin capable of spatially resolved tactile and vibrotactile perception across a wide frequency range [39], while Wang et al. developed a hypersensitive pressure sensor inspired by scorpion slit sensilla that achieved sub-pascal detection thresholds [40]. Integrating such hierarchical surface microstructures or distributed multi-element arrays into a TENG vibration sensor could enable simultaneous broadband frequency discrimination and amplitude quantification, advancing the device from a state-level classifier toward a quantitative vibration analyzer.

Limitations of the present study should also be acknowledged. The on-vehicle test was performed on a single engine model, a single mounting location, and a single session, and generalization across engine types, displacements, and operating environments remains to be verified. The shaker-based benchmark was carried out with a mini electrodynamic shaker (Model 2075E, The Modal Shop, Cincinnati, OH, USA) whose linear operating range constrained the drive amplitude at 150 Hz, and future work using a larger shaker would enable a clean single-amplitude sweep across the full target band. Finally, the absolute voltage predicted by the frequency-amplitude map depends strongly on the mechanical coupling between the device and the vibration source, and the quantitative relationship between map values and on-vehicle voltages will require a dedicated coupling model.

## 5. Conclusions

We have demonstrated a self-powered triboelectric vibration sensor that tracks the complete operating sequence of a running gasoline engine without any external power supply. By co-tuning the air gap and the substrate rigidity across a 3 × 3 device matrix, we identified a clear design window in which a rigid 500 μm glass substrate combined with a 0.5 mm gap simultaneously captures the low-frequency idle and the higher-frequency acceleration components of the engine spectrum. A two-dimensional frequency-amplitude map of the optimized device confirmed that both operating points lie within its sensitive region, and on-vehicle testing resolved all seven predefined operating states through a combination of amplitude envelope and oscillation frequency changes with clear signal-to-noise separation. The comparative behavior of the eight non-optimal devices revealed the characteristic failure modes of overly compliant substrates and oversized gaps, completing a simple and transferable design rule for source-matched TENG vibration sensors. Looking forward, the thin form factor and self-powered operation make the proposed sensor a candidate building block for wireless-ready engine health monitoring. Realizing this potential will require replacing the current PVDF-TrFE and DMF-based process with sustainable, PFAS-free tribo-materials, developing robust packaging against the thermal and chemical environment of an engine compartment, and validating long-term durability over millions of contact-separation cycles.

## Data Availability

Dataset available on request from the authors.

## References

[1] Heywood J.B. (1988). Internal Combustion Engine Fundamentals.

[2] Pulkrabek W.W. (2004). Engineering Fundamentals of the Internal Combustion Engine.

[3] Fleming W.J. (2001). Overview of automotive sensors. IEEE Sens. J..

[4] Zhen X., Wang Y., Xu S., Zhu Y., Tao C. (2012). The engine knock analysis–An overview. Appl. Energy.

[5] Askari H., Khajepour A., Khamesee M.B., Wang Z.L. (2019). Embedded self-powered sensing systems for smart vehicles and intelligent transportation. Nano Energy.

[6] Wu C., Wang A.C., Ding W., Guo H., Wang Z.L. (2019). Triboelectric nanogenerator: A foundation of the energy for the new era. Adv. Energy Mater..

[7] Fan F.-R., Tian Z.-Q., Wang Z.L. (2012). Flexible triboelectric generator. Nano Energy.

[8] Wang Z.L. (2013). Triboelectric nanogenerators as new energy technology for self-powered systems and as active mechanical and chemical sensors. ACS Nano.

[9] Pu X., Guo H., Tang Q., Chen J., Feng L., Liu G., Wang X., Xi Y., Hu C., Wang Z.L. (2018). Rotation sensing and gesture control of a robot joint via triboelectric quantization sensor. Nano Energy.

[10] Yi F., Lin L., Niu S., Yang P.K., Wang Z., Chen J., Zhou Y., Zi Y., Wang J., Liao Q. (2015). Stretchable-rubber-based triboelectric nanogenerator and its application as self-powered body motion sensors. Adv. Funct. Mater..

[11] Wang J., Ding W., Pan L., Wu C., Yu H., Yang L., Liao R., Wang Z.L. (2018). Self-powered wind sensor system for detecting wind speed and direction based on a triboelectric nanogenerator. ACS Nano.

[12] Chen J., Wang Z.L. (2017). Reviving vibration energy harvesting and self-powered sensing by a triboelectric nanogenerator. Joule.

[13] Askari H., Khajepour A., Khamesee M.B., Saadatnia Z., Wang Z.L. (2018). Piezoelectric and triboelectric nanogenerators: Trends and impacts. Nano Today.

[14] Mehamud I., Marklund P., Björling M., Shi Y. (2022). Machine condition monitoring enabled by broad range vibration frequency detecting triboelectric nano-generator (TENG)-based vibration sensors. Nano Energy.

[15] Pang Y., Zhu X., Lee C., Liu S. (2022). Triboelectric nanogenerator as next-generation self-powered sensor for cooperative vehicle infrastructure system. Nano Energy.

[16] Du T., Zuo X., Dong F., Li S., Mtui A.E., Zou Y., Zhang P., Zhao J., Zhang Y., Sun P. (2021). A self-powered and highly accurate vibration sensor based on bouncing-ball triboelectric nanogenerator for intelligent ship machinery monitoring. Micromachines.

[17] Yang W., Chen J., Zhu G., Wen X., Bai P., Su Y., Lin Y., Wang Z.L. (2013). Harvesting vibration energy by a triple-cantilever based triboelectric nanogenerator. Nano Res..

[18] Zhu G., Lin Z.-H., Jing Q., Bai P., Pan C., Yang Y., Zhou Y., Wang Z.L. (2013). Toward large-scale energy harvesting by a nanoparticle-enhanced triboelectric nanogenerator. Nano Lett..

[19] Kim S., Gupta M.K., Lee K.Y., Sohn A., Kim T.Y., Shin K.-S., Kim D., Kim S.K., Lee K.H., Shin H.-J. (2014). Transparent flexible graphene triboelectric nanogenerators. Adv. Mater..

[20] Lee K.Y., Chun J., Lee J.-H., Kim K.N., Kang N.-R., Kim J.-Y., Kim M.H., Shin K.-S., Gupta M.K., Baik J.M. (2014). Hydrophobic sponge structure-based triboelectric nanogenerator. Adv. Mater..

[21] Fang J., Wang X., Lin T. (2011). Electrical power generator from randomly oriented electrospun poly(vinylidene fluoride) nanofibre membranes. J. Mater. Chem..

[22] Zheng J., He A., Li J., Han C.C. (2007). Polymorphism control of poly(vinylidene fluoride) through electrospinning. Macromol. Rapid Commun..

[23] Kuo C.T., Yip M.C., Chiang K.N., Tsou C. (2005). Characterization study of time- and temperature-dependent mechanical behavior of polyimide materials in electronic packaging applications. J. Electron. Mater..

[24] Callister W.D., Rethwisch D.G. (2018). Materials Science and Engineering: An Introduction.

[25] Niu S., Wang S., Lin L., Liu Y., Zhou Y.S., Hu Y., Wang Z.L. (2013). Theoretical study of contact-mode triboelectric nanogenerators as an effective power source. Energy Environ. Sci..

[26] Zi Y., Niu S., Wang J., Wen Z., Tang W., Wang Z.L. (2015). Standards and figure-of-merits for quantifying the performance of triboelectric nanogenerators. Nat. Commun..

[27] Zhang H., Quan L., Chen J., Xu C., Zhang C., Dong S., Lü C., Luo J. (2019). A general optimization approach for contact-separation triboelectric nanogenerator. Nano Energy.

[28] Liu W., Wang Z., Wang G., Liu G., Chen J., Pu X., Xi Y., Wang X., Guo H., Hu C. (2019). Integrated charge excitation triboelectric nanogenerator. Nat. Commun..

[29] Randall R.B. (2011). Vibration-Based Condition Monitoring: Industrial, Aerospace and Automotive Applications.

[30] Qian J., Kim D.S., Lee D.W. (2018). On-vehicle triboelectric nanogenerator enabled self-powered sensor for tire pressure monitoring. Nano Energy.

[31] Lu X., Li H., Zhang X., Gao B., Cheng T. (2022). Magnetic-assisted self-powered acceleration sensor for real-time monitoring vehicle operation and collision based on triboelectric nanogenerator. Nano Energy.

[32] Gao Q., Cheng T., Wang Z.L. (2021). Triboelectric mechanical sensors—Progress and prospects. Extrem. Mech. Lett..

[33] Rodrigues-Marinho T., Brito-Pereira R., Pace G., Tubio C.R., Lanceros-Méndez S., Costa P. (2025). Sustainable polymer materials for triboelectric and hybrid energy harvesting. APL Electron. Devices.

[34] Prat D., Wells A., Hayler J., Sneddon H., McElroy C.R., Abou-Shehada S., Dunn P.J. (2016). CHEM21 selection guide of classical- and less classical-solvents. Green Chem..

[35] Zhao J., Shi Y. (2023). Boosting the durability of triboelectric nanogenerators: A critical review and prospect. Adv. Funct. Mater..

[36] Sobarzo J.C., Pertl F., Balazs D.M., Costanzo T., Sauer M., Foelske A., Ostermann M., Pichler C.M., Wang Y., Nagata Y. (2025). Spontaneous ordering of identical materials into a triboelectric series. Nature.

[37] Wang Z.L. (2020). On the first principle theory of nanogenerators from Maxwell’s equations. Nano Energy.

[38] Wang Z.L., Wang A.C. (2019). On the origin of contact-electrification. Mater. Today.

[39] Du Y., Shen P., Liu H., Zhang Y., Jia L., Pu X., Yang F., Ren T., Chu D., Wang Z.L. (2024). Multi-receptor skin with highly sensitive tele-perception somatosensory. Sci. Adv..

[40] Wang P., Zhang C., Li B., Meng X., Ding Y., Zhang J., Niu S., Han Z., Lin L., Ren L. (2025). Hypersensitive pressure sensors inspired by scorpion mechanosensory mechanisms for near-body flow detection in intelligent robots. Sci. Adv..

